# Preparation and ring-opening reactions of *N*-diphenylphosphinyl vinyl aziridines

**DOI:** 10.3762/bjoc.9.98

**Published:** 2013-05-02

**Authors:** Ashley N Jarvis, Andrew B McLaren, Helen M I Osborn, Joseph Sweeney

**Affiliations:** 1 Department of Chemistry, University of Reading, Reading RG6 6AD, U. K.; 2 Department of Chemical and Biological Sciences, University of Huddersfield, Huddersfield HD1 3DH, U. K.

**Keywords:** aziridines, catalytic, heterocycles, palladium, ring opening

## Abstract

Predominantly (*E*)-*N*-diphenylphosphinyl vinyl aziridines are prepared by a reaction of *N*-diphenylphosphinyl imines with α-bromoallyllithium in the presence of freshly fused ZnCl_2_. These aziridines undergo a ring-opening reaction with a variety of carbon and heteronucleophiles, in good yield, and generally with good regioselectivity.

## Introduction

Vinyl aziridines are useful molecules that have attracted the attention of synthetic chemists for many years. These strained bifunctional heterocycles possess considerable synthetic flexibility and undergo a range of efficient transformations [1–9]. Whilst there has been a recent resurgence in the development of reactions using vinyl aziridines, there have been few contemporary reports of new methods for their synthesis [10–29]. However, a key limitation to the synthetic application of aziridines in general is the nature of the *N*-substituent: most synthetic routes to aziridines deliver *N*-sulfonylated products, and the cleavage of the sulfonamide bond is often challenging. Perhaps for this reason, and despite the plethora of modern reports of the ring opening of vinyl aziridines, synthetic chemists still appear reluctant to employ these potentially valuable synthetic intermediates in target-directed research programmes.

Our group has shown that the diphenylphosphinyl (‘Dpp’) group is a practical alternative to *N*-sulfonylation in aziridine chemistry, due to the ability of the group to activate the ring and the relative ease of its removal at the conclusion of a synthetic manipulation. We report here in full [30] the results of our studies on the synthesis of *N*-Dpp vinyl azidirines, and selected ring-opening reactions of these heterocycles.

## Results and Discussion

### Synthesis of *N*-Dpp vinyl aziridines

#### Initial studies

**Scheme 1:**
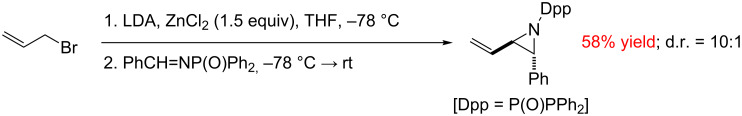
Aza-Darzens synthesis of an *N*-Dpp vinyl aziridine.

We anticipated that the reaction of α-lithio allyl bromide [31–38] with an *N*-Dpp imine would furnish vinyl aziridines by an aza-Darzens-like reaction. This hypothesis was validated by a reaction of this anion with *N*-Dpp benzaldimine at −78 °C in the presence of solid ZnCl_2_, which delivered predominantly (*E*)*-*aziridine **1** (^3^*J* = 2.6 Hz; d.r. = 10:1) [39] in 58% yield (after purification by column chromatography) at the first attempt (Scheme 1).

To our knowledge, this was the first reported direct synthesis of a vinyl aziridine bearing a phosphorus group on nitrogen [40]. Encouraged by this promising result, we next examined the scope of the reaction by using the same anion and *N*-Dpp imines derived from 4-bromo- and 4-fluorobenzaldehyde, furfural and 2,2-dimethylpropionaldehyde. Disappointingly, the yields of aziridines **2**–**5** obtained from these reactions were poor (21–32%), though the diastereoselectivity of the transformation remained good (Table 1). All of the *N*-Dpp imines derived from aromatic aldehydes favoured the formation of (*E*)-vinyl aziridines, with diastereomeric ratios very similar to that of the initial reaction. In particular, the imine derived from furfural yielded only (*E*)-vinyl aziridine **4**. In the case of the vinyl aziridination of 2,2-dimethylpropionaldehyde, an inversion in stereoselectivity was observed, with *Z*–**5** being the dominant partner of the mixture (d.r. = 5:1). (2-Carboxyvinyl)aziridines are particularly useful reagents due, amongst other properties, to their facile conversion into a range of peptidomimetic candidates [41–44]. In an attempt to use our method to deliver these trifunctional aziridines, we reacted the lithium anion derived from methyl (*E*)-3-bromocrotonate with *N*-diphenylphosphinylbenzaldimine. Despite extension optimization studies, the (*E*)-*N*-diphenylphosphinyl-3-[2′-methoxycarbonyl)ethenyl]-2-phenylaziridine (**6**) could only be delivered in mediocre yield (though with excellent diastereoselectivity). Reaction of the same bromocrotonate with *N*-diphenylphosphinyl-4-bromobenzaldimine did not proceed at all when LDA was employed as base. A mixture containing stereochemically inhomogeneous *N*-diphenylphosphinyl-3-[(*E*)-2′-methoxycarbonyl)ethenyl]-2-(4-bromophenyl)aziridine was obtained when other bases were used in place of LDA.

**Table 1:** Aza-Darzens synthesis of *N*-Dpp vinyl aziridines.

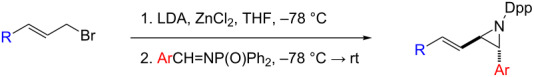					

Ar	R	Product	Method^a^	Yield/%	*Z*:*E*

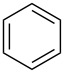	H	**1**	A B	58 71	1:10 1:10
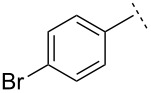	H	**2**	A B	30 62	1:10 1:10
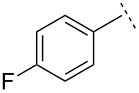	H	**3**	A B	32 64	1:10 1:10
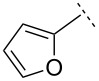	H	**4**	A B	25 45	0:100 0:100
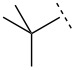	H	**5**	A B	21 50	5:1 5:1
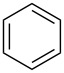	CO_2_Me	**6**	A B	8 25	0:100 0:100
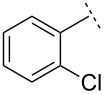	H	**7**	B	67	1:10
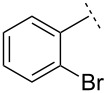	H	**8**	B	55	1:10
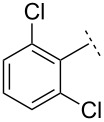	H	**9**	B	68	1:10

^a^Method A: 1 equiv bromide and LDA, 1.5 equiv ZnCl_2_ (fused under Argon); Method B: 1.5 equiv bromide and LDA, 2 equiv ZnCl_2_ fused under vacuum.

#### Optimization studies

We next sought to improve the yields of the vinyl aziridination and thus modified our experimental rubric by carrying out the fusion of ZnCl_2_ under vacuum, rather than at atmospheric pressure. Using this minor modification, the reaction of *N*-diphenylphosphinylbenzaldimine furnished vinyl aziridine **1** in a substantially improved yield (71% versus 58%). Moreover, reactions of the other imines described above were also more efficient. Using the improved method, *N*-Dpp imines derived from 2-chloro-, 2-bromo- and 2,6-dichlorobenzaldehyde also reacted efficiently, yielding vinyl aziridines **7**–**9** in good yields (Table 1) and always in favour of the *E*-isomers (d.r. = 10:1).

#### Mechanism

Some discussion of the mechanism of the reaction is apposite, especially in the light of the single stereochemical anomaly, i.e., the propensity for (*Z*)-vinyl aziridine shown in the reaction of *N*-diphenyphosphinyl-(2,2-dimethyl)ethylimine. To rationalize this divergence, we propose that a closed transition state is in operation in the reactions of aryl imines, and an open TS in the reaction of the pivaldehyde-derived imine.

**Scheme 2:**
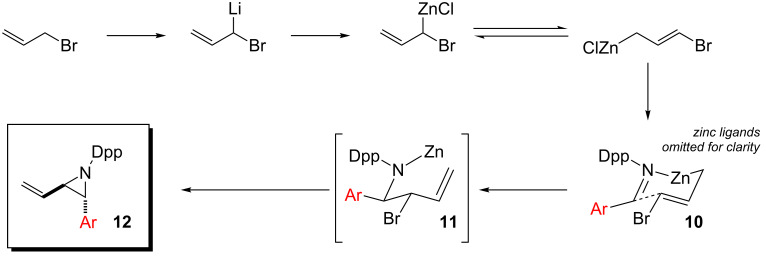
Closed transition state delivers *E*-aziridines.

Thus, in the reaction of aryl imines we presume that lithium–zinc exchange gives an equilibrating allylzinc mixture in which the less-hindered isomer reacts more rapidly, through a chair-like cyclic transition state **10** in which the aryl and bromo substituents are placed in *pseudo*-equatorial positions, with the Dpp-imine. This conformation delivers *anti*-bromoamide **11**, which cyclizes spontaneously to (*E*)-aziridine **12** (Scheme 2).

**Scheme 3:**
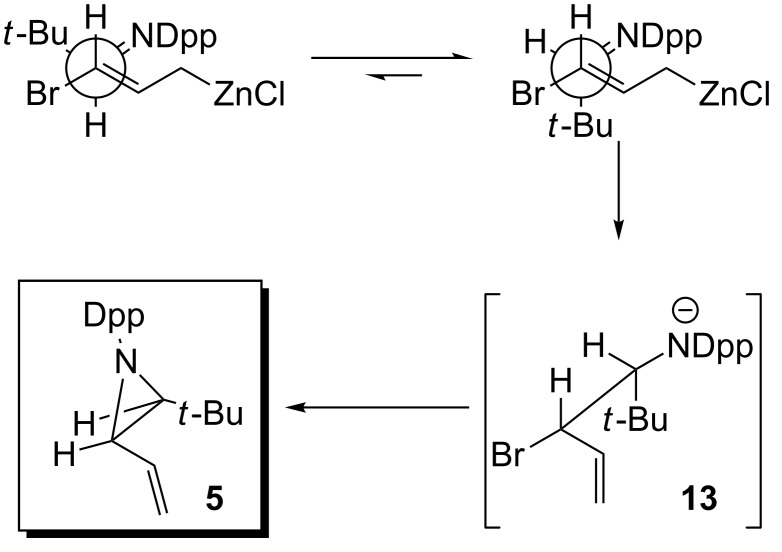
Open transition state leading to (*Z*)-**5**.

In the reaction of *N*-Dpp pivaldimine, we propose that the increased steric demands of the *tert*-butyl group require an open transition state, leading to *syn*-bromo amide anion **13**, which closes to give (*Z*)-aziridine **5** (Scheme 3).

#### Ring opening of *N*-Dpp vinyl aziridines

Having established and optimized the method for the preparation of *N*-Dpp vinyl aziridines, we next turned to an examination of their reactions with a range of nucleophiles, first considering reactions with organometallic reagents. Thus *N*-Dpp vinyl aziridines **1, 2** and **4** reacted with lithio dialkylcuprates with complete regioselectivity, in favour of an S_N_2′ pathway, to give 1,3-disubstituted *N*-Dpp allylamines, generally with good yield (Table 2). In all cases, (*E*)-isomers were obtained. At the time, these data represented the first examples of a 100% regioselective ring opening of simple vinyl aziridines by using lower-order organocuprates. Previously, only 2-(2-carboxyethenyl)aziridines had been shown to undergo ring-opening reactions with a range of nucleophiles [41]. The π-allyl complexes derived from aziridines **1** and **2**, formed in situ by using substoichiometric Pd(PPh_3_)_4_, underwent successful reaction with sodiomalonate and sodium bisphenylsulfonylmethide, again with complete regiocontrol via an S_N_2′-like reaction to give ring-opened products in acceptable yield (Table 2, entries 7, 8, 11, 12).

**Table 2:** S_N_2′-Ring-opening of *N*-Dpp vinyl aziridines by organometallic reagents.

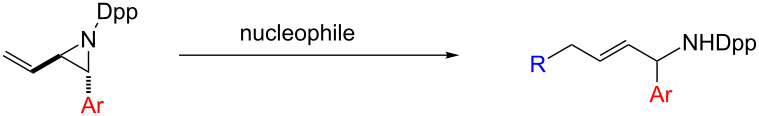					

Entry	Ar	Nucleophile	R	Product	Yield

1	Ph	Me_2_CuLi	Me	**14**	71%
2	Ph	Et_2_CuLi	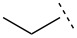	**15**	40%
3	Ph	*n*-Bu_2_CuLi	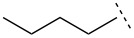	**16**	74%
4	Ph	*s*-Bu_2_CuLi	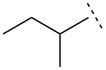	**17**	47%
5	Ph	CH_2_=CH_2_CH_2_MgCl	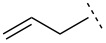	**18**	74%
6	Ph	*t*-Bu_2_CuLi	*t*-Bu	**19**	70%
7	Ph	CH_2_CO_2_Et_2_, Pd(PPh_3_)_4_, NaH	CH(CO_2_Et)_2_	**20**	68%
8	Ph	CH_2_(SO_2_Ph)_2_, Pd(PPh_3_)_4_, NaH	CH(SO_2_Ph)_2_	**21**	62%
9	4-Br-C_6_H_4_	Et_2_CuLi	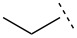	**22**	47%
10	4-Br-C_6_H_4_	*n*-Bu_2_CuLi	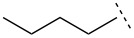	**23**	44%
11	4-Br-C_6_H_4_	CH_2_CO_2_Et_2_, Pd(PPh_3_)4, NaH	CH(CO_2_Et)_2_	**24**	60%
12	4-Br-C_6_H_4_	CH_2_(SO_2_Ph)_2_, Pd(PPh_3_)_4_, NaH	CH(SO_2_Ph)_2_	**25**	57%
13	*t*-Bu	Et_2_CuLi	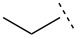	**26**	61%
14	*t*-Bu	*n*-Bu_2_CuLi	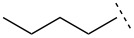	**27**	66%

**Scheme 4:**
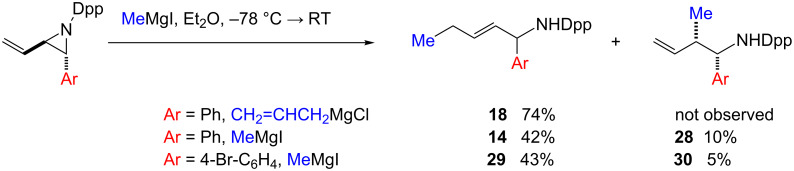
Ring opening by Grignard reagent.

When aziridines **1** and **2** were reacted with methyl magnesium iodide in Et_2_O solution, the previously good regiocontrol we had observed in the ring opening was not maintained: unlike the reaction of allyl magnesium chloride (Table 2, entry 5) a mixture of products arising from nucleophilic attack at both C-2 and C-4 was isolated, with a preference for a S_N_2′-like reaction again observed (Scheme 4).

The reactions of *N*-Dpp vinyl aziridines with heteronucleophiles (tributylstannyl, phenylselanyl and phenylsulfanyl anions) proceeded with variable regioselectivity (Table 3). Thus, bis(tributylstannyl)copper(I) lithium reacted with aziridines **1**, **2** and **4** with complete regiocontrol, as would have been predicted for a copper-based organometallic species, to give *N*-Dpp-4-aminoallylstannanes in good yield (Table 3, entries 1–3). To our knowledge, such allylstannanes had not previously been prepared. Gratified by the good selectivity shown in the ring openings effected by the stannylcuprate, we next examined the reactions of PhSLi and [PhSeBH_3_]Na, predicting that the soft nature of these nucleophiles might also predispose them to react in a manner analogous to that of the stannyl anion. However, our premise was not justified, because these reactions were the most complex we had yet observed, with an inseparable mixture of all three possible ring-opened products being isolated. Two of these compounds arose from S_N_2-like reaction at C-1 and C-2 while the other is produced via an S_N_2′-like reaction at C-4.

**Table 3:** Ring opening of *N*-Dpp vinyl aziridines by heteronucleophiles.

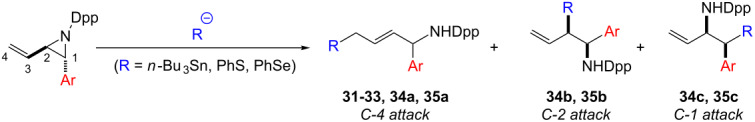						

Entry	Ar	Heteronucleophile	R	Yield/%	Product	C-1:C-2:C-4^a^

1	Ph	(*n*-Bu_3_Sn)_2_CuLi	Bu_3_Sn	65	**31**	C-4 only
2	4-Br-C_6_H_4_	(*n*-Bu_3_Sn)_2_CuLi	Bu_3_Sn	66	**32**	C-4 only
3	*t*-Bu	(*n*-Bu_3_Sn)_2_CuLi	Bu_3_Sn	68	**33**	C-4 only
4	Ph	PhSLi	PhS	75	**34**	19:38:43
5	Ph	[PhSeBH_3_]Na	PhSe	73	**35**	34:51:15

^a^Estimated from ^1^H NMR.

Clearly, the "softness" of the nucleophile does have an effect on the regiochemistry of the ring-opening process (witness the difference in isomer distribution of entries 4 and 5), but there does not yet seem to be any predictability about the reactions. Particularly curious was the poor result obtained when our vinyl aziridines **1**, **2** and **4** were reacted with PhSCu, a reagent which might have been expected to mimic the high selectivity of the reaction with stannylcuprate. Complex mixtures of products were obtained from these reactions.

## Conclusion

We have achieved the first syntheses and ring-opening reactions of *N*-Dpp vinyl aziridines. The ring-opening reactions are selective only when the nucleophilic co-reagent has a natural propensity for S_N_2′-like reaction. A clearer understanding of the features of these reactions is a subject of close scrutiny in our laboratories at this time.

## Experimental

### General methods

All organic solvents were distilled prior to use and all reagents were purified by standard procedures. Light petroleum refers to the fraction boiling in the range 40 to 60 °C. Diethyl ether, THF and DME were distilled from sodium benzophenone ketyl; toluene from sodium; dichloromethane, triethylamine, diisopropylamine and acetonitrile from calcium hydride; and pyridine and diisopropylethylamine from potassium hydroxide.

Melting points were recorded on a Kofler hot-stage apparatus and are uncorrected. IR spectra were recorded on a Perkin Elmer 881 spectrophotometer. Mass spectra were recorded on a VG9090 mass spectrometer or on a Fisons Autospec machine. ^1^H and ^13^C NMR spectra were recorded on a Jeol GX-270 spectrometer or a Jeol L-300 spectrometer. Unless otherwise stated, deuterochloroform was used as the solvent and tetramethylsilane was used as the internal standard. Chemical shifts in ^1^H NMR spectra are expressed as parts per million downfield from tetramethylsilane, and in ^13^C NMR, relative to the internal solvent standard. Coupling constants are quoted in hertz.

Reactions involving chemicals or intermediates sensitive to air and/or moisture were performed under a nitrogen atmosphere in flame- or oven-dried apparatus. Flash column chromatography was performed using Merck Kieselgel 60 or Fluka Kieselgel 60 silica. Analytical thin-layer chromatography (TLC) was performed on precoated Merck Kieselgel 60 F_254_ aluminium backed plates and was visualised under UV conditions at 254 nm and by staining with an acidic ammonium molybdate spray.

### General procedures for the synthesis of *N*-diphenylphosphinyl vinyl aziridines

**Method A**: To freshly distilled diisopropylamine (typically 0.14–0.42 mL, 1–3 mmol, 1.5 equiv) in THF (5 mL) at 0 °C, under argon, was added *n*-butyllithium (2.5 M in hexanes, 1.5 equiv) dropwise. The resulting pale yellow solution was then stirred at 0 °C for 30 min. To a suspension of ZnCl_2_ (1.5 equiv, fused under argon in the reaction vessel) and allyl bromide (1 equiv) in THF (5 mL), under argon, at −78 °C was then added the freshly prepared LDA dropwise. The suspension was stirred at −78 °C for 30 min after which time the *N*-diphenylphosphinylimine (1 equiv) in THF (5 mL) was added dropwise. The solution was further stirred at −78 °C for 1 h and then at room temperature overnight. The solution was diluted with H_2_O (10 mL) and extracted with ethyl acetate (3 × 20 mL). The organic layers were combined, washed with brine (20 mL), dried (MgSO_4_) and filtered, and the solvent was removed in vacuo to leave a yellow oil, which was purified by flash column chromatography on silica gel (typically, ethyl acetate/light petroleum, 1:1, as an eluent).

**Method B**: To freshly distilled diisopropylamine (typically 0.14–0.42 mmol, 1–3 mmol, 2 equiv) in THF (5 mL) at 0 °C, under argon, was added *n*-butyllithium (2.5 M in hexanes, 2 equiv) dropwise. The resulting pale yellow solution was then stirred at 0 °C for 30 min. To a solution of ZnCl_2_ (2 equiv, freshly fused under vacuum), and allyl bromide (1.5 equiv) in THF (5 mL), under argon, at −78 °C was then added the freshly prepared LDA dropwise. The resulting solution was stirred at −78 °C for 30 min, after which time the *N*-diphenylphosphinylimine (1 equiv) in THF (3 mL) was added dropwise over fifteen minutes. The solution was further stirred at −78 °C for 1 h and then at room temperature overnight. The resulting suspension was diluted with H_2_O (10 mL) and extracted with ethyl acetate (3 × 20 mL). The organic layers were combined, washed with brine (20 mL), dried (MgSO_4_) and filtered, and the solvent was removed in vacuo to give a yellow oil, which was purified by flash column chromatography on silica gel (typically, diethyl ether/light petroleum, 3:2, as an eluent).

### General procedures for the ring-opening reactions of *N*-diphenylphosphinyl vinyl aziridines

**Ring-opening reaction with lower-order cuprates:** To dry CuI (5 equiv) in a flame-dried flask, under N_2_, was added diethyl ether (10 mL), and the suspension was degassed. For the preparation of Me_2_CuLi the suspension was then cooled to 0 °C, and MeLi (0.25 mL, 1.4 M in diethyl ether, 0.35 mmol, 1.7 equiv) was added dropwise (the solution becomes yellow after addition of the first equivalent of MeLi and colourless after the addition of the second equivalent). For the preparation of Et_2_CuLi, *n*-Bu_2_CuLi, *s*-Bu_2_CuLi and *t*-Bu_2_CuLi the suspension was cooled to −20 °C and the alkyl lithium (3.5 equiv) was added dropwise affording a dark brown or black suspension. In all cases the solution was stirred for 20 min prior to further cooling to −78 °C. A degassed solution of the vinyl aziridine (1 equiv), in ether (4 mL)/THF (1 mL), was added dropwise, and the solution was stirred at −78 °C for 1 h and then at room temperature for 6 h, and then quenched by the addition of a saturated, aqueous solution of ammonium chloride (15 mL). The mixture was partitioned between ammonium chloride and ethyl acetate, the aqueous layer extracted with ethyl acetate (3 × 15 mL), the organic layers combined and washed with brine (15 mL), dried (MgSO_4_) and filtered, and the solvent removed in vacuo to give a yellow oil. The oil was purified by flash column chromatography on silica gel with ethyl acetate/light petroleum (1:1) as an eluent.

**Palladium-catalyzed ring-opening with malonate:** To diethyl malonate (typically 0.3–0.6 mmol, 1.1 equiv) in THF (1.5 mL), at room temperature, under argon, was added sodium hydride (1.1 equiv), and the colourless solution was left to stir for 30 min. Pd(PPh_3_)_4_ (3 mol %) was added to the reaction mixture and the dark orange suspension was further stirred for twenty five minutes. A solution of vinyl aziridine (1 equiv) in THF (2.5 mL) was added dropwise, and the orange suspension was stirred at room temperature for four hours. The reaction mixture was quenched by the addition of a saturated aqueous solution of ammonium chloride (15 mL). The solution was partitioned between ammonium chloride and ethyl acetate, the aqueous layer extracted with ethyl acetate (3 × 15 mL), the organic layers combined and washed with brine (15 mL), dried (MgSO_4_) and filtered, and the solvent removed in vacuo to give a yellow oil. The oil was purified by flash column chromatography on silica gel (ethyl acetate/light petroleum (1:4); gradient elution to ethyl acetate).

**Palladium-catalyzed ring opening with bis(phenylsulfonyl)methane:** To bis(phenylsulfonyl)methane (typically 0.3–0.7 mmol, 1.1 equiv) in THF (2 mL), at room temperature, under argon, was added sodium hydride (1.1 equiv), and the suspension was left to stir for 30 min. Pd(PPh_3_)_4_ (3 mol %) was added to the reaction mixture, and the dark orange suspension was further stirred for twenty five minutes. A solution of vinyl aziridine (1 equiv) in THF (3 mL) was added dropwise, and the orange suspension was stirred at room temperature for six hours. The reaction mixture was quenched by the addition of a saturated, aqueous solution of ammonium chloride (15 mL). The solution was partitioned between ammonium chloride and ethyl acetate, the aqueous layer extracted with ethyl acetate (3 × 15 mL), the organic layers combined and washed with brine (15 mL), dried (MgSO_4_), filtered and the solvent removed in vacuo to give a yellow oil. The oil was purified by flash column chromatography on silica gel (ethyl acetate/light petroleum (1:4); gradient elution to ethyl acetate).

**Ring-opening reaction with methyl magnesium Grignard:** To magnesium (5 equiv) in diethyl ether (5 mL), under nitrogen, at 0 °C, was added MeI (5.4 equiv) in diethyl ether (1 mL) and the resulting mixture was stirred for 30 min, creating a grey suspension. A solution of the vinyl aziridine (1 equiv) in THF (10 mL) was cooled to −78 °C and the ethereal suspension added dropwise. Immediately, magnesium iodide precipitated as a colourless solid, and the suspension was left to stir at −78 °C for 1 h and then at room temperature for 6–8 hours. The reaction mixture was quenched by the addition of a saturated aqueous solution of ammonium chloride (15 mL). The solution was partitioned between ammonium chloride and ethyl acetate, the aqueous layer extracted with ethyl acetate (3 × 15 mL), the organic layers combined and washed with brine (15 mL), dried (MgSO_4_) and filtered, and the solvent removed in vacuo to give a yellow oil. The oil was purified by flash column chromatography on silica gel with ethyl acetate/light petroleum (1:1) as an eluent.

**Ring-opening reaction with stannyl cuprate:** To freshly distilled diisopropylamine typically (0.4–0.8 mmol, 1 equiv) in THF (5 mL) at 0 °C, under nitrogen, was added *n*-butyllithium (2.5 M in hexanes, 1 equiv) dropwise. The resulting pale yellow solution was then stirred at 0 °C for fifteen minutes, tri-*n*-butyltin hydride (1 equiv) was added dropwise, and the reaction mixture left to stir for a further 15 min. The resulting *n*-Bu_3_SnLi [13] solution was cooled to −20 °C and CuBr·Me_2_S (0.5 equiv) added in one portion. The brown reaction mixture was left to stir for 30 min and subsequently cooled to −78 °C. A solution of the vinyl aziridine (1 equiv) in THF (5 mL) was added dropwise. The solution was stirred at −78 °C for 1 h and left to warm to room temperature overnight, after which time it was quenched by the addition of a saturated aqueous solution of ammonium chloride (15 mL). The solution was partitioned between ammonium chloride and light petroleum, the aqueous layer extracted with light petroleum (3 × 15 mL), the organic layers combined and washed with brine (15 mL), dried (MgSO_4_) and filtered, and the solvent removed in vacuo to give a yellow solid. This was purified by flash column chromatography on silica gel with ethyl acetate/light petroleum (1:1) as an eluent.

**Ring-opening reaction with thiophenolate:** To a solution of thiophenol (0.08 mL, 0.78 mmol) in THF (15 mL), under argon, at −42 °C was added *n*-BuLi (0.34 mL, 2.5 M in hexanes, 0.86 mmol) dropwise. The solution was stirred at −42 °C for 30 min, after which time a solution of aziridine **1** (0.09 g, 0.26 mmol) in THF (5 mL) was added. The solution was allowed to warm to room temperature, and stirring was continued overnight. The solution was then partitioned between water (10 mL) and ethyl acetate (15 mL), the aqueous layer extracted with ethyl acetate (2 × 10 mL), the organic layers washed with brine (15 mL), dried (Na_2_SO_4_) and filtered, and the solvent removed in vacuo to give a yellow oil, which was purified by flash column chromatography on silica gel (ethyl acetate/light petroleum (1:4)) to give **34a** (0.038 g, 32%), as a colourless oil and **34b** and **34c** (0.047 g, 43%, **34b**/**34c** ≈ 2:1, colourless oil) as an inseparable mixture.

## Supporting Information

Supporting Information features detailed experimental data for all compounds.

File 1 Experimental procedures and data.
