# Supplementary material for: Preparation and ring-opening reactions of N-diphenylphosphinyl vinyl aziridines
Source: Beilstein J Org Chem. 2013 May 2;9:852–9. doi: 10.3762/bjoc.9.98 (PMC3678660; doi:10.3762/bjoc.9.98)
Supplement: File 1 — Experimental procedures and data. [file Beilstein_J_Org_Chem-09-852-s001.pdf]

Supporting Information

for

Preparation and ring-opening reactions of *N*-diphenylphosphinyl vinyl aziridines

Ashley N. Jarvis[1], Andrew B. McLaren[1], Helen M. I. Osborn[1] and Joseph Sweeney*[1,2]

Address: [1]Department of Chemistry, University of Reading, Reading RG6 6AD, U. K. [2]Current address: Department of Chemical and Biological Sciences, University of Huddersfield, Huddersfield HD1 3DH, U. K.

Email: Joseph Sweeney - j.b.sweeney@hud.ac.uk

* Corresponding author

Experimental procedures and data

***N*-(Diphenylphosphinyl)-1-phenyl-2-vinylaziridine (1)** [1]:

Following Method A, LDA (1.5 equiv), prepared from diisopropylamine (0.14 mL, 0.98 mmol) and *n*-butyllithium (0.39 mL, 2.5 M in hexanes, 0.98 mmol) was added to a suspension of $ZnCl_2$ (0.13 g, 0.98 mmol) and allyl bromide (0.06 mL, 0.66 mmol) in THF and the resulting yellow solution was combined with *P*,*P*-diphenyl-*N*-(benzylidene)phosphinic amide (0.2 g, 0.66 mmol) to afford a yellow oil. The oil was purified by column chromatography to give:

(*E*)-**1**, as a colourless solid (0.11 g, 48%); R_f 0.5 (ethyl acetate); mp 158 °C; (Found: C, 76.3; H, 6.0; N, 4.0. $C_{22}H_{20}NOP$ requires C, 76.5; H, 5.8; N, 4.1);

$\nu_{max}(CCl_4)/cm^{-1}$ 3400, 3061, 2927, 1437, 1240, 1127, 1093; δ_H (270 MHz, $CDCl_3$) 3.17 (1H, ddd, *J* = 2.6, 9.9, 12.4), 3.91 (1H, dd, *J* = 2.6, 15.8) 5.13 (1H, dd, *J* = 0.9, 9.9), 5.24 (1H, dd, *J* = 0.9, 17.1), 6.30 (1H, ddd, *J* = 9.9, 9.9, 17.1) 7.26-7.49 and 7.82–7.99 (15H, m); δ_C (67.5 MHz, $CDCl_3$) 43.03 and 52.49, 120.15, 126.08 127.70, 128.08, 128.18, 128.37, 128.46, 128.53, 128.53, 131.48, 131.67; δ_P (161.7 MHz, $CDCl_3$) 29.81; *m*/*z* (CI) 346 ($[MH]^+$, 100%), 201 (26%), 144 (35%), 79 (20%) (Found 346.1367. $C_{22}H_{21}NOP$ requires 346.1361);
(*Z*)-**1**, as a colourless oil (0.01 g, 5%): R_f 0.4 (ethyl acetate); mp 160–161 °C; (Found: C, 76.2; H, 5.85; N, 3.72. $C_{22}H_{20}NOP$ requires C, 76.5; H, 5.8; N, 4.06); $\nu_{max}(CCl_4)/cm^{-1}$ 3061, 2926, 1438, 1209, 1126, 1110, 727, 697; δ_H (270 MHz, $CDCl_3$) 3.50–3.61 (1H, m), 4.06 (1H, dd, *J* = 6.2, 16.5) 5.13 (1H, dd, *J* = 2.2, 9.3), 5.32 (1H, dd, *J* = 2.2, 17.2), 5.44 (1H, ddd, *J* = 7.7, 9.3, 17.2), 7.21–7.53 and 7.89–8.01 (15H, m); δ_C (75.5 MHz, $CDCl_3$) 41.70, 43.40, 120.73 and 127.49–131.98; *m*/*z* (CI) 346 ($[MH]^+$, 100%), 201 (22), 144 (31) (Found: $[MH]^+$, 346.1353. $C_{22}H_{21}NOP$ requires $[MH]^+$, 346.1361).

Following Method B, LDA (2 equiv), prepared from diisopropylamine (0.27 mL, 1.97 mmol) and *n*-butyllithium (0.79 mL, 2.5 M in hexanes, 1.97 mmol) was added to a solution of $ZnCl_2$ (0.27 g, 1.97 mmol) and allyl bromide (0.12 mL, 1.48 mmol) in THF and the resulting yellow solution was combined with *P*,*P*-diphenyl-*N*-(benzylidene)phosphinic amide (0.3 g, 0.98 mmol) to produce a yellow oil. The oil was purified by column chromatography to give **1** (0.24 g, 71%) as a colourless solid. Analytical data corresponded exactly with those obtained using Method A.

_N_-(Diphenylphosphinyl)-1-(4'-bromophenyl)-2-vinylaziridine (2)

Following Method A, LDA (1.5 equiv), made from diisopropylamine (0.27 mL, 1.96 mmol) and *n*-butyllithium (0.78 mL, 2.5 M in hexanes, 1.96 mmol) was added to a suspension of $ZnCl_2$ (0.26 g, 1.96 mmol) and allyl bromide (0.11 mL, 1.31 mmol) in THF and the resulting yellow solution was combined with *P*,*P*-diphenyl-*N*-(4'-bromobenzylidene)phosphinic amide (0.5 g, 1.31 mmol) to produce a yellow oil. The oil was purified by column chromatography to give:

(E)-**2** (0.15 g, 27%) as a colourless solid: R_f 0.5 (ethyl acetate); mp 179–180 °C; (Found: C, 62.5; H, 4.8; N, 3.35. $C_{22}H_{19}BrNOP$ requires C, 62.3; H, 4.5; N, 3.3); $\nu_{max}(CCl_4)/cm^{-1}$ 3400, 3060, 2930, 1200, 1126, 1070; δ_H (300 MHz, $CDCl_3$) 3.13 (1H, ddd, J = 2.8, 9.9, 12.3), 3.86 (1H, dd, J = 2.8, 15.8), 5.14 (1H, dd, J = 0.9, 9.9), 5.25 (1H, dd, J = 0.9, 17.0), 6.28 (1H, ddd, J = 9.9, 9.9, 17.0), 7.16–7.48 and 7.80–7.96 (14H, m); δ_C (67.5 MHz, $CDCl_3$) 42.49 and 52.43, 120.46, 121.57, 127.80, 128.24, 128.43, 128.59, 128.78, 131.45, 131.61, 131.70, 131.83, 131.99, 133.80, 136.30; δ_P (121.6 MHz, $CDCl_3$) 30.09; m/z (CI) 426 (100%) 424 ($[MH]^+$, 99.6), 346 (19), 201 (48), 79 (5);

(Z)-**2**, as a colourless oil (0.02 g, 3%): R_f 0.4 (ethyl acetate); IR data as for E-isomer; δ_H (300 MHz, $CDCl_3$) 3.50–3.60 (1H, m), 4.03 (1H, dd, J = 6.2, 16.5), 5.16 (1H, dd, J = 3.0, 8.8), 5.29–5.46 (2H, 2 × m), 7.18–7.52 and 7.86–7.99 (14H, m); δ_C (75.5 MHz, $CDCl_3$) 41.68 and 43.35 (2 × d, J = 6.2 6.8), 121.10, 128.44, 128.60, 129.23, 131.30, 131.41, 131.54, 131.59, 131.71, 131.88, 132.07, 132.11, 132.94, 133.83; m/z (CI) 426 (100%) (424 ($[MH]^+$, 99.6), 346 (28), 201 (41), 79 (7) (Found: $[MH]^+$, 424.0466. $C_{22}H_{20}BrNOP$ requires $[MH]^+$, 424.0456).

_N_-(Diphenylphosphinyl)-1-(4'-fluorophenyl)-2-vinylaziridine (3)

Following Method A, LDA (1.5 equiv), made from diisopropylamine (0.26 mL, 1.86 mmol) and n-butyllithium (0.74 mL, 2.5 M in hexanes, 1.86 mmol) was added to a suspension of $ZnCl_2$ (0.25 g, 1.86 mmol) and allyl bromide (0.10 mL, 1.24 mmol) in THF and the resulting yellow solution was combined with P,P-diphenyl-N-(4'-fluorobenzylidene)phosphinic amide (0.4 g, 1.24 mmol) to produce aziridine **3**, a yellow oil, as a 10:1 E:Z mixture of isomer, as judged by ^1H NMR. The oil was purified by column chromatography to give:

(E)-**3** as a colourless solid (0.14 g, 29%); R_f 0.4 [(diethyl ether/light petroleum, 7:3)]; mp 141–143 °C; $\nu_{max}(CCl_4)/cm^{-1}$ 3055, 2987, 1266; δ_H (400 MHz, $CDCl_3$) 3.06 (1H, ddd, J = 2.9, 9.9, 12.4), 3.80 (1H, dd, J = 2.9, 15.8), 5.06 (1H, br d, J = 10.1), 5.17 (1H, dd, J = 1.1,16.9), 6.20 (1H, ddd, J = 10.1, 10.1, 16.9), 6.91 (2H, m), 7.17–7.43 and 7.74–7.88 (12H, m); δ_C (100 MHz, $CDCl_3$) 42.41 (d, J = 5.5) and 52.26 (d, J = 7.3), 115.26, 115.48, 120.27, 127.62, 128.21, 128.33, 131.44, 131.54, 131.70,

132.10, 132.78, 133.35, 134.077, 161.09; *m*/*z* (EI) ([M]$^+$ 363, 25%), 201 (68), 162 (100), 122 (47), 77 (27) (Found: [M]$^+$, 363.1194. $C_{22}H_{19}FNOP$ requires [M]$^+$, 363.1188);

The *Z*-isomer could not be isolated.

(*E*)-*N*-(Diphenylphosphinyl)-1-(2'-furyl)-2-vinylaziridine (4)

Following Method A, LDA (1.5 equiv), made from diisopropylamine (0.31 mL, 2.34 mmol) and *n*-butyllithium (0.94 mL, 2.5 M in hexanes, 2.34 mmol) was added to a suspension of $ZnCl_2$ (0.32 g, 2.34 mmol) and allyl bromide (0.13 mL, 1.56 mmol) in THF and the resulting brown solution was combined with P,P-Diphenyl-N-(2'-furanmethylene)phosphinic amide (0.46 g, 1.56 mmol) to produce a dark brown oil. The oil was purified by column chromatography to give:

(*E*)-**4**, as a colourless oil (0.13 g, 25%); R_f 0.65 (ethyl acetate); ν_{max}(film)/cm^{-1} 3061, 1438, 1205, 1151, 1125, 1110, 723, 694; δ_H (270 MHz, $CDCl_3$) 3.57 (1H, ddd, *J* = 2.8, 9.9, 12.4), 3.90 (1H, dd, *J* = 2.8, 15.3), 5.17 (1H, dd, *J* = 1.0, 9.9), 5.33 (1H, dd, *J* = 1.0, 17.1), 6.19 (1H, ddd, *J* = 9.9, 9.9, 17.1), 6.25 (1H, dd, *J* = 0.4, 3.3), 6.31 (1H, dd, *J* = 1.8, 3.1), 7.32–7.50 and 7.80–7.91 (11H, m); δ_C (75.5 MHz, $CDCl_3$) 37.42 (d, *J* = 5.6), 48.32 (d, *J* = 8.0), 108.53, 110.633, 120.60, 128.19, 128.23, 128.41, 131.52, 131.65, 131.75, 133.79, 142.30, 150.10 (d, *J* = 5.0); *m*/*z* (CI) 336 ([MH]$^+$, 100%), 258 (8), 231 (10), 201 (24), 134 (36) (Found: [MH]$^+$, 336.1156. $C_{20}H_{19}NO_2P$ requires [MH]$^+$, 336.1153).

***N*-(Diphenylphosphinyl)-1-*tert*-butyl-2-vinylaziridine (5)**

Following Method A, LDA (1.5 equiv), made from diisopropylamine (0.37 mL, 2.63 mmol) and *n*-butyllithium (1.05 mL, 2.5 M in hexanes, 2.63 mmol) was added to a suspension of $ZnCl_2$ (0.36 g, 2.63 mmol) and allyl bromide (0.15 mL, 1.75 mmol) in THF and the resulting yellow solution was combined with *P*,*P*-diphenyl-*N*-(*tert*-butylmethylene)phosphinic amide (0.5 g, 1.75 mmol) to produce a yellow oil. The oil was purified by column chromatography to give:

(*E*)-**5**, as a colourless oil (0.02 g, 3%): R_f 0.36 [(ethyl acetate/light petroleum, 1:1)]; ν_{max}(CCl_4)/cm^{-1} 3396, 3060, 2965, 1438, 1207, 1125, 1109, 727, 695 (Ar); δ_H (270 MHz, $CDCl_3$) 0.80 (9H, s), 2.79 (1H, dd, *J* = 3.2, 16.7), 3.01-3.10 (1H, m), 5.01 (1H,

dd, *J* = 1.4, 10.1), 5.22 (1H, dd, *J* = 1.4, 17.1), 6.10 (1H, ddd, *J* = 10.1, 10.1, 17.1), 7.30–7.47 and 7.84–7.97 (10H, m); δ_C (67.5 MHz, $CDCl_3$) 26.35, 30.89, 46.19, 52.28, 120.26, 129.16-135.22; *m*/*z* (CI) 326 ($[MH]^+$, 100%), 298 (23), 286 (49), 201 (40), 124 (60) (Found: $[MH]^+$, 326.1675. $C_{20}H_{25}NOP$ requires $[MH]^+$, 326.1674);

Z-(5) as a colourless oil (0.10 g, 18%): R_f 0.3 [(ethyl acetate-light petroleum, 1:1)]; $\nu_{max}(CCl_4)/cm^{-1}$ 3438, 3062, 2969, 1438, 1209, 1125, 716 (Ar); δ_H (270 MHz, $CDCl_3$) 0.76 (9H, s), 2.64 (1H, dd, *J* = 6.4, 17.7), 3.20-3.28 (1H, m), 5.19 (1H, dd, *J* = 1.3, 10.5), 5.31 (1H, dd, *J* = 1.3, 17.2), 5.96 (1H, ddd, *J* = 8.2, 10.5, 17.2) 7.27–7.50 and 7.89–7.95 (10H, m); δ_C (67.5 MHz, $CDCl_3$) 29.04, 32.49, 42.88 and 50.65, 120.45, 129.14-135.14; *m*/*z* (CI) 326 ($[MH]^+$, 100%), 286 (17), 270 (19), 201 (23), 79 (4) (Found: $[MH]^+$, 326.1674. $C_{20}H_{25}NOP$ requires $[MH]^+$, 326.1674).

(*E*)-*N*-(Diphenylphosphinyl)-1-phenyl-[(*E*)-2-(methoxycarbonyl)ethenyl]aziridine (6)

Following Method A, LDA (1.5 equiv), made from diisopropylamine (0.34 mL, 2.46 mmol) and *n*-butyllithium (0.98 mL, 2.5 M in hexanes, 2.46 mmol) was added to a suspension of $ZnCl_2$ (0.33 g, 2.46 mmol) and methyl 4-bromocrotonate (0.20 mL, 1.75 mmol) in THF and the resulting yellow solution was combined with *P*,*P*-diphenyl-*N*-(benzylidene)phosphinic amide (0.5 g, 1.75 mmol) to produce a yellow oil. The oil was purified by column chromatography to give:

(*E*)-**6**, as a colourless oil (0.05 g, 8%); R_f 0.5 (ethyl acetate); $\nu_{max}(film)/cm^{-1}$ 3062, 2927, 1729, 1211, 1124, 1110, 697; δ_H (270 MHz, $CDCl_3$) 3.19 (1H, ddd, *J* = 2.4, 9.5, 11.9), 3.71 (3H, s), 4.03 (1H, dd, *J* = 2.4, 15.6, 5.89 (1H, d, *J* = 15.6), 7.26–7.54 (10H, m), 7.85–7.93 (5H, m); δ_C (67.5 MHz, $CDCl_3$) 29.66, 44.21 and 51.67, 125.02, 126.15, 128.15, 128.30, 128.50, 128.62, 131.32, 131.45, 131.54, 131.66, 131.86, 132.05, 133.16, 133.77, 165.51; *m*/*z* (CI) 404 ($[MH]^+$, 100%), 372 (24%), 344 (15%), 218 (8%), 202 (20%) (Found: $[MH]^+$, 404.1415. $C_{24}H_{23}NO_3P$ requires $[MH]^+$, 404.1416).

N-(Diphenylphosphinyl)-1-(2'-chlorophenyl)-2-vinylaziridine (7)

Following Method B, LDA (2 equiv), prepared from diisopropylamine (0.16 mL, 1.18 mmol) and *n*-butyllithium (0.47 mL, 2.5 M in hexanes, 1.18 mmol) was added to a solution of $ZnCl_2$ (0.16 g, 1.18 mmol) and allyl bromide (0.08 mL, 0.89 mmol) in THF and the resulting yellow solution was combined with the *P*,*P*-diphenyl-*N*-(2'-chlorobenzylidene)phosphinic amide (0.2 g, 0.59 mmol) to produce aziridine (**7**), a yellow oil, as a 10:1 *E*:*Z* mixture of isomers, as judged by ^{1}H NMR. The oil was purified by column chromatography to give*:*

(*E*)-**7**, as a colourless solid (0.14 g, 61%): R_f 0.5 (ethyl acetate); mp 130 °C; ν_{max}(CCl_4)/cm^{-1} 3448, 3056, 2961, 1267, 1115; δH (400 MHz, $CDCl_3$) 3.00 (1H, ddd, *J* = 2.8, 9.9, 12.5), 4.20 (1H, dd, *J* = 2.8, 15.4), 5.08 (1H, br d, *J* = 10.2), 5.24 (1H,br d, *J* = 17.0), 6.25 (1H, ddd, *J* = 9.9, 9.9, 17.0) 7.08-7.43 and 7.81-7.93 (14H, m); δ_C (75.5 MHz, $CDCl_3$) 41.75, 52.18, 120.96 and 127.04–132.16; *m*/*z* (CI) ([MH]$^+$ 380, 100%), 344 (25), 218 (59), 203 (48) 180 (37) (Found: [MH]$^+$, 380.0958. $C_{22}H_{20}ClNOP$ requires [MH]$^+$, 380.0971).

The *Z*-isomer could not be isolated.

N-(Diphenylphosphinyl)-1-(2'-bromophenyl)-2-vinylaziridine (8)

Following Method B, LDA (2 equiv), prepared from diisopropylamine (0.22 mL, 1.6 mmol) and *n*-butyllithium (0.63 mL, 2.5 M in hexanes, 1.6 mmol) was added to a solution of $ZnCl_2$ (0.21 g, 1.6 mmol) and allyl bromide (0.10 mL, 1.17 mmol) in THF and the resulting yellow solution was combined with *P*,*P*-diphenyl-*N*-(2'-bromophenylmethylene)phosphinic amide (0.3 g, 0.78 mmol) to produce a yellow oil. The oil was purified by column chromatography to give:

(*E*)-**8**, as a colourless oil (0.08 g, 50%): R_f 0.5 (ethyl acetate); ν_{max}(film)/cm^{-1} 3055, 2987, 1203, 1127, 1110, 706, 666; δ_H (400 MHz, $CDCl_3$) 2.97 (1H, ddd, *J* = 2.9, 9.5, 12.5), 4.16 (1H, dd, *J* = 2.9, 15.4), 5.08 (1H, br d, *J* = 9.9), 5.21 (1H,dd, *J* = 1.1, 17.0), 6.25 (1H, ddd, *J* = 9.9, 9.9, 17.0), 6.99–7.46 and 7.82–7.94 (14H, m); δ_C (100 MHz, $CDCl_3$), .43.53 and 51.80 (d, *J* = 7.3), 120.54, 123.69, 126.98, 127.31, 128.28, 128.41, 128.90, 131.52, 131.65, 131.83, 131.90, 132.38, 133.95 134.02; *m*/*z* (CI) 426 (42%) 424 ([MH]$^+$, 44), 344 (34), 201 (100), 77 (37);

(Z)-**8**, as a colourless oil, (0.01 g, 5%): R_f 0.4 (ethyl acetate); δ_H (400 MHz, $CDCl_3$), 3.56–3.63 (1H, m), 4.11 (1H, dd, J = 6.2, 15.8), 5.03 (1H, dd, J = 2.2, 9.9), 5.20 (1H, ddd, J = 7.3, 9.9, 17.2), 5.28 (1H, dd, J = 2.2, 17.2), 7.01–7.48 and 7.82–7.93 (14H, m); m/z (CI) 426 (100%) 424 ($[MH]^+$, 99.6), 344 (40), 201 (78), 77 (28) (Found: $[MH]^+$, 424.0466. $C_{22}H_{20}BrNOP$ requires $[MH]^+$, 424.0469).

_N_-(Diphenylphosphinyl)-1-(2',6'-dichlorophenyl)-2-vinylaziridine (9)

Following Method B, LDA (2 equiv), prepared from diisopropylamine (0.30 mL, 2.14 mmol) and *n*-butyllithium (0.86 mL, 2.5 M in hexanes, 2.14 mmol) was added to a solution of $ZnCl_2$ (0.29 g, 2.14 mmol) and allyl bromide (0.14 mL, 1.61 mmol) in THF and the resulting yellow solution was combined with *P*,*P*-diphenyl-*N*-(2',6'-dichlorobenzylidene)phosphinic amide (0.4 g, 1.07 mmol) to produce a yellow oil. The oil was purified by column chromatography to give:

(E)-**9**, as a colourless oil (0.27 g, 62%): R_f 0.55 (ethyl acetate); ν_{max}(film)/cm^{-1} 3054, 2984, 1440, 1266, 1198, 1110 740, 703; δ_H (400 MHz, $CDCl_3$) 3.50 (1H, br. dd, J = 10.2, 12.5), 4.05 (1H, br. d, J = 14.7), 4.96 (1H, br. d, J = 10.2), 5.16 (1H, br. d, J = 16.9), 6.01 (1H, m) 6.98–7.46 and 7.75–7.95 (13H, m); δ_C (100 MHz, $CDCl_3$) 42.58 and 49.12, 120.73, 127.82–136.16; m/z (CI) 414 ($[MH]^+$, 72%), 378 (52), 212 (100), 201 (64), 77 (27); *Z*-(9) (0.03 g, 6%): R_f 0.5(ethyl acetate); ν_{max}(CCl_4)/cm^{-1} 3062, 2988, 1433, 1124, 1109, 736, 696; δ_H (400 MHz, $CDCl_3$) 3.55-3.62 (1H, m), 4.18 (1H, dd, J = 5.9, 16.1), 5.09 (1H, br. d, J = 9.2), 5.08–5.37 (2H, m), 7.06–7.56 and 7.83–8.04 (13H, m); δ_C (75.5 MHz, $CDCl_3$) 41.51 and 42.28, 120.19 and 127.98-135.86; m/z (CI) 414 ($[MH]^+$, 31%), 378 (48), 218 (30), 201 (100), 77 (24).

Reactions of vinyl aziridines (1), (2) and (5)

With lower-order cuprate reagents

To dry CuI (5 equiv) in a flame-dried flask, under N_2, was added diethyl ether (10 mL) and the suspension degassed. For the preparation of Me_2CuLi the suspension was then cooled to 0 °C and MeLi (0.25 mL, 1.4 M in diethyl ether, 0.35 mmol, 1.7 equiv) added dropwise. The solution becomes yellow after addition of the first equivalent of MeLi and colourless after the addition of the second equivalent. For the preparation

of Et_2CuLi, *n*-Bu_2CuLi, *sec*-Bu_2CuLi and *tert*-Bu_2CuLi the suspension was cooled to −20 °C, and the alkyl lithium (3.5 equiv) was added dropwise affording a dark brown or black suspension. In all cases the solution was stirred for 20 min prior to further cooling to −78 °C. A degassed solution of the vinylaziridine (1 equiv), in ether (4 mL)/THF (1 mL), was added dropwise. The solution was stirred at −78 °C for 1 h and then at room temperature for 6 h, and then quenched by the addition of a saturated, aqueous solution of ammonium chloride (15 mL). The solution was partitioned between ammonium chloride and ethyl acetate, the aqueous layer extracted with ethyl acetate (3 × 15 mL), the organic layers combined and washed with brine (15 mL), dried ($MgSO_4$), filtered and the solvent removed in vacuo to give a yellow oil. The oil was purified by flash column chromatography on silica gel with ethyl acetate/light petroleum (1:1) as an eluent.

(*E*)-1-(Diphenylphosphinamido)-1-phenylpent-2-ene (14)

By following the general method described above, reaction of vinylaziridine **1** (0.04 g, 0.12 mmol) with Me_2CuLi, prepared from CuI (0.033 g, 0.17 mmol) and MeLi (0.25 mL, 1.4 M in diethyl ether, 0.35 mmol), afforded **14** (0.029 g, 69%) as a colourless solid, R_f 0.44 (ethyl acetate); mp 175-176 °C; $\nu_{max}(CCl_4)/cm^{-1}$ 3124, 2930, 2860, 1438, 1255, 1209, 1122, 715, 696; δ_H (270 MHz, $CDCl_3$) 0.91 (3H, t, *J* = 7.3), 1.93–2.04 (2H, m), 3.27 (1H, dd, *J* = 6.2, 8.8), 4.76-4.85 (1H, m) 5.53 (1H, dt, *J* = 5.5, 15.4), 5.64 (1H, dd, *J* = 5.9, 15.4), 7.20–7.52 and 7.78-7.97 (15H, m); δ_C (67.5 MHz, $CDCl_3$) 13.22, 25.28, 56.94, 126.72, 127.16, 128.24, 128.43, 128.50, 130.59, 131.54, 131.73, 131.92, 132.08, 132.18, 132.33, 133.35, 133.96; *m*/*z* (CI) 362 ($[MH]^+$, 40%), 218 (61), 160 (11), 145 (73), 79 (100) (Found: $[MH]^+$, 362.1671. $C_{23}H_{25}NOP$ requires $[MH]^+$, 362.1674).

(*E*)-1-(Diphenylphosphinamido)-1-phenylhex-2-ene (15)

By following the general method described above, reaction of vinylaziridine **1** (0.04 g, 0.12 mmol) and Et_2CuLi, prepared from CuI (0.033 g, 0.17 mmol) and EtLi (≈0.35 mmol in 2.5 mL of ether), afforded **15** (0.02 g, 47%) as a colourless solid, R_f 0.40 (ethyl acetate); mp 126–127 °C; (Found: C, 76.75; H, 7.0; N, 3.5. $C_{24}H_{26}NOP$ requires C, 76.8; H, 7.0; N, 3.7); $\nu_{max}(CCl_4)/cm^{-1}$ 3380, 2928, 1438, 1210, 1124,

698; δ_H (270 MHz, $CDCl_3$) 0.86 (3H, t, *J* = 7.3), 1.26-1.37 (2H, m), 1.91–1.99 (2H, m), 3.26 (1H, dd, *J* = 6.2, 8.8), 4.75-4.84 (1H, m, 5.50-5.54 (1H, m), 5.64 (1H, dd, *J* = 6.2, 15.4), 7.21–7.52 and 7.78–7.96 (15H, m); δ_C (67.5 MHz, $CDCl_3$) 13.66, 22.17, 34.17, 56.90, 126.94, 127.16, 128.24, 128.43, 131.57, 131.73, 131.86, 132.08, 132.18, 132.24, 133.83; *m*/*z* (CI) 376 ($[MH]^+$, 70), 218 (25), 201 (66), 174 (100),129 (18) (Found: $[MH]^+$, 376.1830. $C_{24}H_{27}NOP$ requires $[MH]^+$, 376.1830).

(*E*)-1-(Diphenylphosphinamido)-1-phenyloct-2-ene (16)

By following the general method described above, reaction of vinylaziridine **1** (0.04 g, 0.12 mmol) with *n*-Bu_2CuLi, prepared from CuI (0.033 g, 0.17 mmol) and *n*-BuLi (0.15 mL, 2.5 M in hexanes, 0.35 mmol), afforded **16** (0.035 g, 74%) as a colourless oil, R_f 0.42 (ethyl acetate); ν_{max}(film)/cm^{-1} 3379, 2928, 2856, 1438, 1211, 1123, 697; δ_H (270 MHz, $CDCl_3$) 0.84–0.92 and 1.25–1.32 (9H, m), 1.93–1.98 (2H, m), 3.25 (1H, dd, *J* = 6.2, 8.8), 4.76–4.86 (1H, m), 5.51–5.55 (1H, m), 5.64 (1H, dd, *J* = 6.2, 15.4), 7.24–7.48 and 7.79–7.96 (15H, m); δ_C (67.5 MHz, $CDCl_3$) 14.05, 22.49, 28.71, 31.44, 32.11, 56.94, 126.97, 127.19, 128.27, 128.46, 131.64, 131.77, 131.92, 132.11, 132.27, 132.37, 132.59; *m*/*z* (CI) 404 ($[MH]^+$, 100%), 218 (50), 202 (61), 187 (25), 144 (55) (Found: $[MH]^+$, 404.2141. $C_{26}H_{31}NOP$ requires $[MH]^+$, 404.2143).

(*E*)-1-(Diphenylphosphinamido)-1-phenyl-5-methylhept-2-ene (17)

By following the general method described above, reaction of vinylaziridine **1** (0.04 g, 0.12 mmol) and *sec*-Bu_2CuLi, prepared from CuI (0.033 g, 0.17 mmol) and *sec*-BuLi (0.31 mL, 1.3 M in hexanes, 0.35 mmol), afforded **17** (0.02 g, 43%) as a colourless oil, R_f 0.43 (ethyl acetate); ν_{max}(film)/cm^{-1} 3379, 2930, 2856, 1210, 1127, 697; δ_H (270 MHz, $CDCl_3$) 0.82–0.91 and 1.25–1.34 (9H, m), 1.93–1.98 (2H, m), 3.24 (1H, dd, *J* = 6.2, 8.8), 4.76–4.86 (1H, m, 5.51-5.55 (1H, m), 5.64 (1H, dd, *J* = 6.2, 15.4), 7.26–7.49 and 7.78–7.98 (15H, m); *m*/*z* (CI) 404 ($[MH]^+$, 10), 218 (95), 202 (41), 187 (36), 79 (49) (Found: $[MH]^+$, 404.2139. $C_{26}H_{31}NOP$ requires $[MH]^+$, 404.2143).

(*E*)-1-(Diphenylphosphinamido)-1-phenylhept-2,6-ene (18)

By following the general method described above, reaction of vinylaziridine **1** (0.2 g, 0.58 mmol) and allylmagnesium chloride (2.0 M in THF) afforded **18** (0.10g, 46%) as a colourless solid, R_f 0.4 (ethyl acetate); $\nu_{max}(CCl_4)/cm^{-1}$ 3379, 2925, 2852, 1438, 1210, 1122, 740, 698 δ_H (300 MHz, $CDCl_3$) 2.06–2.08 (4H, br m), 3.26 (1H, dd, *J* = 6.2, 9.5), 4.76–4.84 (1H, m, 4.95 (1H, br dd, *J* = 1.5, 10.2), 5.02 (1H, v. br. d, *J* = 1.5), 5.53 (1H, dt, *J* = 6.2, 15.2), 5.65–5.83 (2H, m), 7.20–7.53 and 7.79–7.96 (15H, m); δ_C (100 MHz, $CDCl_3$) 31.49, 33.19, 56.92, 114.79, 126.98, 127.26, 128.33, 128.50, 128.55, 131.82, 132.14, 132.22, 132.35, 142.93; δ_P (121.6 MHz, $CDCl_3$) 22.66; *m*/*z* (CI) 388 ($[MH]^+$, 100%), 346 (10), 306 (9), 218 (40), 201 (11) (Found: $[MH]^+$, 388.1822. $C_{25}H_{27}NOP$ requires $[MH]^+$, 388.1830).

(*E*)-1-(Diphenylphosphinamido)-1-phenyl-5,5-dimethylhex-2-ene (19)

By following the general method described above, reaction of vinylaziridine **1** (0.04 g, 0.12 mmol) and *tert*-Bu_2CuLi, prepared from CuI (0.033g, 0.17 mmol) and *tert*-BuLi (0.31 mL, 1.3 M in hexanes, 0.35 mmol), afforded **19** (0.032 g, 68%) as a colourless oil, R_f 0.43 (ethyl acetate); $\nu_{max}(film)/cm^{-1}$ 3378, 3060, 2928, 2856, 1436, 1210, 1125, 697; the ^1H NMR spectrum of **19** exhibited duplication of signals due to rotameric isomers: δ_H (270 MHz, $CDCl_3$) 0.75 and 0.85 (9H, 2 × s, rotamers A and B), 1.64 and 1.73 (2H, 2 × ddd, *J* = 1.7, 7.9, 14.7, rotamer A or B) and 1.89 (2H, br. d, *J* = 7.0, rotamer A or B), 3.25 (1H, dd, *J* = 6.4, 8.8), 4.82 (1H, ddd, *J* = 6.4, 9.7, 9.7, PhC<u>H</u>, rotamer A or B), 5.10 (1H, ddd (app. q), *J* = 9.7, PhC<u>H</u>, rotamer A or B), 5.48–5.60 and 5.63–5.75 (2H, 2 x m and PhCH), 7.19–7.53 and 7.79–7.99 (15H, m); δ_C (270 MHz, $CDCl_3$) 29.21 and 29.68 (rotamers A and B), 30.76 and 31.01 ($Me_3\underline{C}$, rotamers A and B), 41.21, 46.70, 51.97 and 56.96 ($Me_3C\underline{C}H_2$ and Ph<u>C</u>H, rotamers A and B), 126.96, 127.18, 127.97, 128.32, 128.51, 129.53, 131.72, 132.01, 132.17, 132.29, 132.37, 132.55, 133.59, 134.10; *m*/*z* (CI) 404 ($[MH]^+$, 100%), 346 (13), 218 (36), 202 (31), 187 (24) (Found: $[MH]^+$, 404.2137. $C_{26}H_{31}NOP$ requires $[MH]^+$, 404.2143).

(*E*)-1-(Diphenylphosphinamido)-1-(4'-bromophenyl)-hex-2-ene (22)

By following the general method described above, reaction of vinylaziridine **2** (0.04 g, 0.10 mmol) and Et_2CuLi, prepared from CuI (0.033 g, 0.17 mmol) and EtLi (≈0.35 mmol in 2.5 mL of ether), afforded **22** (0.022 g, 47%) as a colourless solid, R_f 0.38 (ethyl acetate); mp 94–95 °C; $\nu_{max}(CCl_4)/cm^{-1}$ 3376, 2960, 2928, 2855, 1438, 1210, 1123, 698 δ_H (270 MHz, $CDCl_3$) 0.86 (3H, t, *J* = 7.3), 1.25–1.38 (2H, m), 1.92–2.0 (2H, m), 3.25 (1H, dd, *J* = 6.2, 8.8), 4.75–4.84 (1H, m), 5.50–5.54 (1H, m), 5.64 (1H, dd, *J* = 6.1, 15.4), 7.21–7.52 and 7.78–7.96 (14H, m); δ_C (75.5 MHz, $CDCl_3$) 13.73, 22.21, 34.22, 56.92, 126.98, 127.21, 128.31, 128.47, 128.52, 131.80, 132.14, 132.27, 132.34; *m*/*z* (CI) 454 ($[MH]^+$, 0.7%), 376 (M − Br, 100), 218 (80), 201 (19), 187 (20), 159 (33). (Found: $[MH]^+$, 376.1812, $C_{24}H_{27}NOP$ requires $[MH]^+$, 376.1830).

(*E*)-1-(Diphenylphosphinamido)-1-(4'-bromophenyl)-oct-2-ene (23)

By following the general method described above, reaction of vinylaziridine **2** (0.045 g, 0.12 mmol) and *n*-Bu_2CuLi, prepared from CuI (0.034 g, 0.18 mmol) and *n*-BuLi (0.15 mL, 2.5 M in hexanes, 0.38 mmol), afforded **23** (0.025 g, 44%) as a colourless solid, R_f 0.39 (ethyl acetate); mp 103–104 °C; $\nu_{max}(CCl_4)/cm^{-1}$ 3380, 2928, 2857, 1439, 1211, 1122, 709, 697; δ_H (270 MHz, $CDCl_3$) 0.85–0.95 and 1.25–1.32 (9H, m), 1.94–1.99 (2H, m), 3.26 (1H, dd, *J* = 6.4, 8.8), 4.74-4.82 (1H, m), 5.46–5.58 (1H, m), 5.64 (1H, dd, *J* = 5.9, 15.4), 7.26–7.53 and 7.76–7.95 (14H, m); δ_C (67.5 MHz, $CDCl_3$) 14.01, 22.43, 28.65, 31.38, 32.08, 56.30, 128.34, 128.50, 128.81, 131.51, 131.83, 131.99, 132.15, 132.24, 132.30, 133.07; *m*/*z* (CI) 484 (22%), 482 ($[MH]^+$, 24), 404 (9), 218 (49), 85 (100) (Found: $[MH]^+$, 481.1168. $C_{26}H_{29}BrNOP$ requires $[MH]^+$, 481.1170).

(*E*)-1-(Diphenylphosphinamido)-1-*tert*-butylhex-2-ene (26)

By following the general method described above, reaction of vinylaziridine **5** (0.04 g, 0.12 mmol) and Et_2CuLi, prepared from CuI (0.035g, 0.18 mmol) and EtLi (≈0.35 mmol in 2.5 mL of ether), afforded **26** (0.027 g, 61%) as a colourless oil, R_f 0.40 (ethyl acetate); $\nu_{max}(film)/cm^{-1}$ 3400, 2929, 2870, 1438, 1214, 1122, 1110, 710, 697; δ_H (270 MHz, $CDCl_3$) 0.86 (3H, t, *J* = 7.3), 0.92 (9H, s,), 1.26-1.37 (2H, m),

1.86–1.97 (2H, m), 2.84 (1H, dd, J = 7.1, 9.9), 3.18–3.26 (1H, m), 5.18-5.28 (1H, m), 5.34 (1H, dd, J = 7.7, 15.4), 7.38–7.48 and 7.80–7.96 (10H, m); δ_C (67.5 MHz, $CDCl_3$) 13.70, 22.27, 26.59), 29.66, 34.62, 62.94, 128.01, 128.24, 128.43, 129.23, 131.54, 131.80, 131.92, 132.62, 132.75, 132.97; *m/z* (CI) 356 ($[MH]^+$, 100%), 298 (33), 218 (18), 201 (8), 149 (8), 85 (15) (Found: $[MH]^+$, 356.2128. $C_{22}H_{31}NOP$ requires $[MH]^+$, 356.2143).

(*E*)-1-(Diphenylphosphinamido)-1-*tert*-butyloct-2-ene (27)

By following the general method described above, reaction of vinylaziridine **5** (0.04g, 0.12 mmol) and n-Bu_2CuLi, prepared from CuI (0.035 g, 0.18 mmol) and n-BuLi (0.16 mL, 2.5 M in hexanes, 0.39 mmol), afforded **27** (0.031 g, 66%) as a colourless oil, R_f 0.40 (ethyl acetate); ν_{max}(film)/cm^{-1} 3404, 2961, 2858, 1211, 1123, 697 δ_H (270 MHz, $CDCl_3$) 0.85–0.90 and 1.24–1.30 (9H, m), 0.92 (9H, s,), 1.93–1.98 (2H, m), 2.84 (1H, dd, J = 7.1, 9.9), 3.20–3.30 (1H, m), 5.18–5.28 (1H, m), 5.34 (1H, dd, J = 7.7, 15.4), 7.38–7.48 and 7.80–7.94 (10H, m); δ_C (67.5 MHz, $CDCl_3$) 14.05, 22.46, 26.59, 28.78, 31.41, 32.20, 34.66, 62.87, 127.99, 128.18, 128.24, 128.43, 128.78, 129.00, 131.54, 132.30, 132.78, 133.89; *m/z* (CI) 384 ($[MH]^+$, 100%), 326 (48), 306 (18), 218 (17), 201 (9) (Found: $[MH]^+$, 384.2463. $C_{24}H_{35}NOP$ requires $[MH]^+$, 384.2456).

Palladium-catalyzed ring-opening reactions

With malonate

To diethyl malonate (typically 0.3–0.6 mmol, 1.1 equiv) in THF (1.5 mL), at room temperature, under argon, was added sodium hydride (1.1 equiv), and the colourless solution was left to stir for thirty minutes. $Pd(PPh_3)_4$ (3 mol %) was added to the reaction mixture and the dark orange suspension was further stirred for twenty five minutes. A solution of vinylaziridine (1 equiv) in THF (2.5 mL) was added dropwise, and the orange suspension was stirred at room temperature for four hours. The reaction mixture was quenched by the addition of a saturated aqueous solution of ammonium chloride (15 ml). The solution was partitioned between ammonium chloride and ethyl acetate, the aqueous layer extracted with ethyl acetate (3 × 15 mL), the organic layers combined and washed with brine (15 mL), dried ($MgSO_4$),

and filtered, and the solvent removed in vacuo to give a yellow oil. The oil was purified by flash column chromatography on silica gel (ethyl acetate/light petroleum (1:4); gradient elution to ethyl acetate).

Ethyl 2-ethoxycarbonyl-6-(diphenylphosphinamido)-6-phenyl-4-hexenoate (20)

By following the general method described above vinylaziridine **1** (0.2 g, 0.58 mmol) and a suspension of diethyl malonate (0.1 mL, 0.64 mmol), sodium hydride (0.015 g, 0.64 mmol) and $Pd(PPh_3)_4$ (0.020 g, 3 mol %) were combined to give **20** (0.2 g, 68%) as a colourless solid, R_f 0.38 (ethyl acetate); mp 103–104 °C; (Found: C, 68.85; H, 6.3; N, 2.5. $C_{29}H_{32}NOP$ requires C, 68.9; H, 6.4; N, 2.8); $\nu_{max}(CCl_4)/cm^{-1}$ 3370, 3059, 2982, 1750, 1735, 1438, 1210, 1122, 717, 695 δ_H (300 MHz, $CDCl_3$) 1.21 (6H, 2 × t (coincidental), *J* = 7.1), 2.59 (1H, 2 × dd (br) (coincidental), *J* = 7.4), 3.25 (1H, dd, *J* = 6.2, 9.5), 3.33 (1H, t, *J* = 7.4), 4.10–4.16 (4H, m), 4.73–4.82 (1H, m, 5.48–5.57 (1H, m), 5.78 (1H, dd, *J* = 6.1, 15.4), 7.23–7.49, 7.79–7.91 (15H, m); δ_C (75.5 MHz, $CDCl_3$) 14.05, 31.26, 51.74, 56.60, 61.41 (d, *J* = 1.8), 126.84, 127.25, 127.38, 128.33, 128.42, 128.51, 128.58, 131.54, 131.83, 131.93, 132,03, 132.13, 132.19, 135.04, 168.80 (d, *J* = 1.3); *m/z* (CI) 506 ($[MH]^+$, 100%), 492 (16), 460 (11), 346 (20), 218 (58), 201 (16) (Found: $[MH]^+$, 506.2096. $C_{29}H_{33}NO_5P$ requires $[MH]^+$, 506.2096).

Ethyl 2-ethoxycarbonyl-6-(diphenylphosphinamido)-6-(4'-bromophenyl)-4-hexenoate (24)

By following the general method described above vinylaziridine **1** (0.095 g, 0.23 mmol) and a suspension of diethyl malonate (0.04 mL, 0.25 mmol), sodium hydride (7 mg, 0.25 mmol) and $Pd(PPh_3)_4$ (8 mg, 3 mol %) were combined to give **24** (0.078 g, 60%) as a colourless oil, R_f 0.4 (ethyl acetate); $\nu_{max}(film)/cm^{-1}$ 3379, 3059, 2981, 2934, 1751, 1735, 1437, 1215, 1121, 722, 696 δ_H (300 MHz, $CDCl_3$) 1.21 (6H, 2 × t (coincidental), *J* = 7.1), 2.60 (1H, 2 × dd (br) (coincidental), *J* = 7.3), 3.27 (1H, dd, *J* = 6.2, 9.7), 3.34 (1H, t, *J* = 7.3), 4.11–4.18 (4H, m), 4.69–4.77 (1H, m), 5.48–5.57 (1H, m), 5.75 (1H, dd, *J* = 6.1, 15.2), 7.14–7.16, 7.37–7.49, 7.63–7.90 (14H, m); δ_C (75.5 MHz, $CDCl_3$) 14.01, 31.19, 51.62, 55.98, 61.42, 121.27, 127.73, 128.36, 128.52, 128.58, 131.57, 131.89, 131.99, 132.12, 132.27, 134.43, 141.40, 168.69;

m/*z* (CI) 586 (100%), 584 ([MH]$^+$, 95%), 506 (14), 424 (16), 384 (18), 218 (90), 201 (24) (Found: [MH]$^+$, 584.1202. $C_{29}H_{32}BrNO_5P$ requires [MH]$^+$, 584.1201).

With bis(phenylsulfonyl)methane

To bis(phenylsulfonyl)methane (typically 0.3–0.7 mmol, 1.1 equiv) in THF (2 mL), at room temperature, under argon, was added sodium hydride (1.1 equiv), and the suspension was left to stir for thirty minutes. $Pd(PPh_3)_4$ (3 mol %) was added to the reaction mixture and the dark orange suspension was further stirred for twenty five minutes. A solution of vinylaziridine (1 equiv) in THF (3 mL) was added dropwise, and the orange suspension was stirred at room temperature for six hours. The reaction mixture was quenched by the addition of a saturated, aqueous solution of ammonium chloride (15 mL). The solution was partitioned between ammonium chloride and ethyl acetate, the aqueous layer extracted with ethyl acetate (3 × 15 mL), the organic layers combined and washed with brine (15 mL), dried ($MgSO_4$), and filtered, and the solvent removed in vacuo to give a yellow oil. The oil was purified by flash column chromatography on silica gel (ethyl acetate/light petroleum [1:4]; gradient elution to ethyl acetate).

1,1-Bis(phenylsulfonyl)-5-(diphenylphosphinamido)-5-phenyl-pent-3-ene (21)

By following the general method described above vinylaziridine **1** (0.2 g, 0.58 mmol) and a suspension of bis(phenylsulfonyl)methane (0.19 g, 0.64 mmol), sodium hydride (0.015 g, 0.64 mmol) and $Pd(PPh_3)_4$ (0.020 g, 3 mol %) were combined to give **21** (0.23 g, 62%) as a colourless oil, R_f 0.42 (ethyl acetate); ν_{max}(film)/cm^{-1} 3369, 3052, 2986, 1438, 1200, 1123, 1109, 737; δ_H (300 MHz, $CDCl_3$) 2.83–2.88 (2H, m), 3.30 (1H, dd, *J* = 6.2, 9.9), 4.52 (1H, t, *J* = 6.0), 4.68–4.76 (1H, m, 5.56-5.65 (1H, m), 5.69 (1H, dd, *J* = 5.0, 15.4), 7.21–7.64 and 7.80–7.94 (25H, m); δ_C (75.5 MHz, $CDCl_3$), 28.34, 56.42, 83.09, 125.84, 126.93, 127.58, 128.42, 128.48, 128.58, 128.73, 129.62, 131.83, 131.99, 132.04, 132.87, 133.54, 134.57, 136.29, 137.83, 141.95 (d, *J* = 6.3); *m*/*z* (CI) 642 ([MH]$^+$, 100%), 500 (11), 440 (7), 346 (10), 218 (22), 201 (15), 143 (45) (Found: [MH]$^+$, 642.1532. $C_{35}H_{33}NO_5PS_2$ requires [MH]$^+$, 642.1538).

1,1-Bis(phenylsulfonyl)-5-(4'-bromophenyl)-5-(diphenylphosphinamido)-pent-3-ene (25)

By following the general method described above vinylaziridine **2** (0.1 g, 0.24 mmol) and a suspension of bis(phenylsulfonyl)methane (0.08 g, 0.26 mmol), sodium hydride (8 mg, 0.26 mmol) and $Pd(PPh_3)_4$ (9 mg, 3 mol %) were combined to give **25** (0.097 g, 57%) as a colourless solid, R_f 0.12 (ethyl acetate/light petroleum 1:1); mp 187–188 °C; $\nu_{max}(CCl_4)/cm^{-1}$ 3371, 3057, 2923, 1438, 1200, 1123, 1109, 716, 681; δ_H (300 MHz, $CDCl_3$) 2.90 (1H, 2 × dd (br) (coincidental), *J* = 6.1), 3.26 (1H, dd, *J* = 6.2, 9.9), 4.50 (1H, t, *J* = 6.0), 4.66-4.74 (1H, m), 5.56-5.65 (1H, m), 5.72 (1H, dd, *J* = 5.3, 15.4), 7.11–7.14, 7.39–7.69 and 7.78–7.94 (24H, m); δ_C (75.5 MHz, $CDCl_3$) 28.37, 55.77, 82.92, 125.82, 128.46, 128.56, 128.64, 128.72, 128.82, 129.13, 129.61, 129.64, 131.76, 132.04, 132.17, 132.32, 134.65, 140.93; *m*/*z* (CI) 722 (48%), 720 ($[MH]^+$, 41%), 419 (36), 363 (20), 297 (12), 218 (100), 201 (18), 143 (38) (Found: $[MH]^+$, 720.0654. $C_{35}H_{32}BrNO_5PS_2$ requires $[MH]^+$, 720.0643).

With Grignard reagents

To magnesium (5 equiv) in diethyl ether (5 mL), under nitrogen, at 0 °C, was added MeI (5.4 equiv) in diethyl ether (1 mL), and the resulting mixture was stirred for thirty minutes, creating a grey suspension. A solution of the vinylaziridine (1 equiv) in THF (10 mL) was cooled to −78 °C and the ethereal suspension added dropwise. Immediately, magnesium iodide precipitated as a colourless solid and the suspension was left to stir at −78 °C for one hour and then at room temperature for 6–8 hours. The reaction mixture was quenched by the addition of a saturated aqueous solution of ammonium chloride (15 mL). The solution was partitioned between ammonium chloride and ethyl acetate, the aqueous layer extracted with ethyl acetate (3 × 15 mL), the organic layers combined and washed with brine (15 mL), dried ($MgSO_4$), and filtered, and the solvent removed in vacuo to give a yellow oil. The oil was purified by flash column chromatography on silica gel with ethyl acetate/light petroleum (1:1) as an eluent.

(*E*)-1-(Diphenylphosphinamido)-1-phenylpent-2-ene (14) and 1-(diphenylphosphinamido)-1-phenyl-2-methylbut-3-ene (28)

By following the general method described above, reaction of vinylaziridine **1** (0.095 g,0.28 mmol) and MeMgI, prepared from Mg (0.033 g, 1.38 mmol) and MeI (0.09 mL, 1.49 mmol), afforded a colourless solid as an inseparable mixture of **14** and **28** (0.070 g, 52%). 1H NMR indicated the product to be a 4.2:1 mixture of **14** and **28**. Data for **28**: δ_H (300 MHz, $CDCl_3$) 1.00 (3H, d, *J* = 7.0), 2.56 (1H, m), 3.38 (1H, dd, *J* = 5.9, 9.1), 4.09 (1H, m) 5.09 (1H, br d, *J* = 17.2), 5.16 (1H, dd, *J* = 1.1, 10.0), 5.81 (1H, ddd, *J* = 7.3, 10.0, 17.2), 7.20–7.50 and 7.79–7.97 (15H, m); (Found: $[MH]^+$, 362.1669. $C_{23}H_{25}NOP$ requires $[MH]^+$, 362.1674).

(*E*)-1-(Diphenylphosphinamido)-1-(4'-bromophenyl)-pent-2-ene (29) and 1-(diphenylphosphinamido)-1-(4'-bromophenyl)-2-methylbut-3-ene (30)

By following the general method described above, reaction of vinylaziridine **2** (0.29 g, 0.69 mmol) and MeMgI, prepared from Mg (0.082 g, 3.43 mmol) and MeI (0.23 mL, 3.7 mmol), afforded:

29 (0.13 g, 43%) as a colourless solid, R_f 0.38 (ethyl acetate); $\nu_{max}(CCl_4)/cm^{-1}$ 3400, 2964, 2928, 2855, 1438, 1210, 1123, 719, 697; δ_H (270 MHz, $CDCl_3$) 0.92 (3H, t, *J* = 7.3), 1.95–2.04 (2H, m), 3.30 (1H, dd, *J* = 6.2, 9.2), 4.71–4.80 (1H, m), 5.50–5.54 (1H, m), 5.64 (1H, dd, *J* = 6.1, 15.4), 7.21–7.52 and 7.78–7.96 (14H, m); δ_C (75.5 MHz, $CDCl_3$) 13.27, 25.12, 56.34, 121.05, 128.32, 128.35, 130.27, 131.52, 131.61, 131.72, 131.81, 131.92, 132.04, 132.17, 132.32, 133.44; *m*/*z* (CI) 442 (25%), 440 ($[MH]^+$, 25), 362 (30), 218 (28), 85 (56), 57 (100) (Found: $[MH]^+$, 440.0761. $C_{23}H_{24}BrNOP$ requires $[MH]^+$, 440.0779), and

30 as a colourless solid (16 mg, 5%), δ_H (300 MHz, $CDCl_3$) 0.97 (3H, d, *J* = 6.8), 2.50 (1H, m), 3.40 (1H, dd, *J* = 5.5, 8.1), 4.03 (1H, m, 5.10 (1H, br d, *J* = 17.2), 5.17 (1H, dd, *J* = 1.1, 9.5), 5.77 (1H, ddd, *J* = 7.7, 9.5, 17.2, 7.26–7.50 and 7.79–7.94 (14H, m); (Found: $[MH]^+$, 440.0780. $C_{23}H_{24}BrNOP$ requires $[MH]^+$, 440.0779).

With stannyl cuprates

To freshly distilled diisopropylamine typically (0.4–0.8 mmol, 1 equiv) in THF (5 mL) at 0 °C, under nitrogen, was added *n*-butyllithium (2.5 M in hexanes, 1 equiv),

dropwise. The resulting pale yellow solution was then stirred at 0 °C for fifteen minutes and tri-*n*-butyltin hydride (1 equiv) was added dropwise and the reaction mixture left to stir for a further 15 minutes. The resulting *n*-Bu_3SnLi solution was cooled to −20 °C and CuBr·Me_2S (0.5 equiv) added in one portion. The brown reaction mixture was left to stir for thirty minutes and subsequently cooled to −78 °C. A solution of the vinylaziridine (1 equiv) in THF (5 mL) was added dropwise. The solution was stirred at −78 °C for one hour and left to warm to room temperature overnight, after which time it was quenched by the addition of a saturated aqueous solution of ammonium chloride (15 mL). The solution was partitioned between ammonium chloride and light petroleum, the aqueous layer extracted with light petroleum (3 × 15 mL), the organic layers combined and washed with brine (15 mL), dried ($MgSO_4$), and filtered, and the solvent removed in vacuo to give a yellow solid. This was purified by flash column chromatography on silica gel with ethyl acetate/light petroleum (1:1) as an eluent.

(*E*)-1-(Diphenylphosphinamido)-1-phenyl-4-tri-n-butylstannylbut-2-ene (31)

By following the general method described above, tri-*n*-butyltin hydride (0.21 mL, 0.78 mmol) was added to a solution of LDA [diisopropylamine (0.1 mL, 0.78 mmol)/*n*-butyllithium (0.31 mL, 2.5 in hexanes, 0.78 mmol)] in THF (5 mL) and CuBr·Me_2S (0.08 g, 0.39 mmol) was added at −20 °C. Vinylaziridine **1** (0.27 g, 0.78 mmol) and $[(Bu_3Sn)_2Cu]Li$ thus formed, were combined to afford **31** (0.33 g, 65%) as a colourless waxy solid, R_f 0.39 (ethyl acetate/light petroleum 1:1); $\nu_{max}(CCl_4)/cm^{-1}$ 3378, 3059, 2982, 1648, 1597, 1438, 1211, 1123, 713, 697 δ_H (300 MHz, $CDCl_3$) 0.79–0.94 and 1.19–1.44 (27H, m, 1.66 (2H, d, *J* = 7.0), 3.23 (1H, dd, *J* = 6.1, 9.3), 4.73–4.81 (1H, m, 5.58 (1H, dd, *J* = 5.9, 15.1), 5.62–5.73 (1H, m), 6.99–7.30, 7.96-8.11 (15H, m); δ_C (75.5 MHz, $CDCl_3$) 9.21, 13.65, 14.24, 27.26, 29.03, 57.12, 126.84, 126.91, 127.37, 128.18, 128.30, 128.35, 128.43, 131.65, 132.09, 132.16, 132.21, 132.29; *m*/*z* (CI) 638 ($[MH]^+$, 87%), 580 (56), 450 (30), 346 (30), 218 (100) 201 (30) (Found: $[MH]^+$, 638.2565. $C_{34}H_{49}NOPSn$ requires $[MH]^+$, 638.2573).

(*E*)-1-(Diphenylphosphinamido)-1-(4'-bromophenyl)-4-tri-*n*-butylstannylbut-2-ene (32)

By following the general method described above, tri-*n*-butyltin hydride (0.11 mL, 0.4 mmol) was added to a solution of LDA [diisopropylamine (0.06 mL, 0.4 mmol)/*n*-butyllithium (0.16 mL, 2.5 M in hexanes, 0.4 mmol)] in THF (5 mL) and CuBr·Me_2S (0.04 g, 0.2 mmol) was added at −20 °C. Vinylaziridine **2** (0.17 g, 0.4 mmol) and $[(Bu_3Sn)_2Cu]Li$ thus formed were combined to afford **33** (0.19 g, 66%) as a colourless solid, R_f 0.5 (ethyl acetate/light petroleum 1:1); mp 60–61°C; (Found: C, 57.2; H, 6.4; N, 1.7. $C_{34}H_{47}BrNOPSn$ requires C, 57.1; H, 6.6; N, 2.0); $\nu_{max}(CCl_4)/cm^{-1}$ 3374, 3059, 2956, 2923, 1658, 1591, 1438, 1210, 1122, 717, 670 δ_H (300 MHz, $CDCl_3$) 0.76–0.87 and 1.19–1.44 (27H, m), 1.67 (2H, d, *J* = 8.3), 3.18 (1H, dd, *J* = 5.8, 9.2), 4.69–4.77 (1H, m) 5.43 (1H, dd, *J* = 6.1, 15.2), 5.49-5.60 (1H, m), 7.11-7.14 , 7.34–7.51 and 7.75–7.91 (14H, m); δ_C (75.5 MHz, $CDCl_3$) 9.29, 13.68, 14.35, 27.30, 29.07, 56.62, 120.79, 126.65, 126.72, 128.27, 128.37, 128.43, 128.54, 128.73, 131.37, 132.09, 132.22, 132.90, 142.50; *m*/*z* (CI) 716 ($[MH]^+$, 52%), 658 (38), 450 (44), 336 (26), 218 (100), 201 (24), 130 (84) (Found: $[MH]^+$, 716.1669. $C_{34}H_{48}BrNOPSn$ requires $[MH]^+$, 716.1679).

(*E*)-1-(Diphenylphosphinamido)-1-*tert*-butyl-4-tri-n-butylstannylbut-2-ene (33)

By following the general method described above, tri-*n*-butyltin hydride (0.08 mL, 0.29 mmol) was added to a solution of LDA [diisopropylamine (0.04 mL, 0.29 mmol)/*n*-butyllithium (0.12 mL, 2.5 M in hexanes, 0.29 mmol)] in THF (5 mL) and CuBr·Me_2S (0.03 g, 0.15 mmol) was added at −20 °C. Vinyl aziridine **5** (0.095 g, 0.29 mmol) and $[(Bu_3Sn)_2Cu]Li$ thus formed were combined to give **34** (0.12 g, 68%) as a white waxy solid, R_f 0.50 (ethyl acetate/light petroleum 3:1); (Found: C, 62.3; H, 8.1; N, 2.0. $C_{32}H_{53}NOPSn$ requires C, 62.4; H, 8.4; N, 2.3); $\nu_{max}(CCl_4)/cm^{-1}$ 3374, 3060, 2957, 2926, 1651, 1552, 1438, 1211, 1122, 736, 697; δ_H (300 MHz, $CDCl_3$) 0.79–0.92 and 1.23–1.48 (36H, m), 1.64 (2H, d, *J* = 10.1,), 2.79 (1H, dd, *J* = 7.2, 9.5), 3.19-3.28 (1H, m), 5.13 (1H, dd, *J* = 8.4, 15.2), 5.37 (1H, ddd, *J* = 6.8, 10.1, 15.2), 7.41–7.48 and 7.81–7.93 (10H, m); δ_C (75.5 MHz, $CDCl_3$) 9.19, 13.62, 14.02, 26.70, 27.34, 29.12, 63.37, 124.64, 128.01, 128.18, 128.25, 128.42, 131.45, 131.52, 131.56, 131.84, 131.96, 132.71, 132.77, 132.83; *m*/*z* (CI) 618 ($[MH]^+$, 50%), 560

(100), 450 (5), 336 (8), 218 (9) (Found: $[MH]^+$, 618.2898. $C_{33}H_{53}NOPSn$ requires $[MH]^+$, 618.2887).

With thiophenolate anion

1-(Diphenylphosphinamido)-1-phenyl-4-(phenylthio)-but-2-ene (34a), 1-(diphenylphosphinamido)-1-phenyl-2-(phenylthio)-but-3-ene (34b) and 2-(diphenylphosphinamido)-1-phenyl-1-(phenylthio)-but-3-ene (34c)

To a solution of thiophenol (0.08 mL, 0.78 mmol) in THF (15 mL), under argon, at −42 °C was added *n*-BuLi (0.34 mL, 2.5 M in hexanes, 0.86 mmol) dropwise. The solution was stirred at −42 °C for thirty minutes, after which time a solution of aziridine **1** (0.09 g, 0.26 mmol) in THF (5 mL) was added. The solution was allowed to warm to room temperature and stirring continued overnight. The solution was then partitioned between water (10 mL) and ethyl acetate (15 mL), the aqueous layer extracted with ethyl acetate (2 × 10 mL), the organic layers washed with brine (15 mL), dried (Na_2SO_4), and filtered, and the solvent removed in vacuo to give a yellow oil, which was purified by flash column chromatography on silica gel [ethyl acetate/light petroleum (1:4)] to give **34a** (0.038 g, 32%) as a colourless oil and **34b** and **34c** (0.047 g, 43%, **34b**:**34c** ≈ 2:1, colourless oil) as an inseparable mixture.

Data for **34a**: R_f 0.15 [ethyl acetate-light petroleum (1:1)]; ν_{max}(film)/cm^{-1} 3379, 3062, 2926, 1213, 1124, 1110, 714, 698 δ_H (270 MHz, $CDCl_3$) 3.11 (1H, dd, *J* = 6.2, 10.1), 3.51 (2H, d, *J* = 6.8), 4.69–4.77 (1H, m, 5.53–5.63 (1H, m), 5.72 (1H, dd, *J* = 5.7, 15.2), 7.10–7.51 and 7.75–7.90 (20H, m); δ_C (67.5 MHz, $CDCl_3$) 36.07, 54.58, 125.29–132.48; *m/z* (CI) 456 ($[MH]^+$, 92%), 346 (52), 306 (70), 239 (100), 201 (26), 111 (25) (Found: $[MH]^+$, 456.1551. $C_{28}H_{27}NOPS$ requires $[MH]^+$, 456.1551).

Data for **34b** & **34c**: R_f 0.14 [ethyl acetate/light petroleum (1:1)]; ν_{max}(film)/cm^{-1} 3348, 3062, 1209, 1124, 1110, 697.

Data for **34b**: δ_H (270 MHz, $CDCl_3$) 4.09 (1H, dd, *J* = 4.9, 8.7), 4.13 (1H, dd, *J* = 6.4, 10.2), 4.47 (1H, ddd, *J* = 4.9, 10.2, 10.2), 4.91–4.97 (2H, br. m), 5.54 (1H, ddd, *J* = 8.7, 9.7, 17.2), 7.03–7.43 and 7.62–7.84 (20H, m).

Data for **34c**: δ_H (270 MHz, $CDCl_3$) 3.66 (1H, dd, *J* = 6.3, 11.0), 4.07–4.13 (1H, m), 4.71 (1H, d, *J* = 3.7) 5.12–5.21 (2H, br. m), 5.74 (1H, ddd, *J* = 5.6, 10.8, 16.5), 7.12–7.52 and 7.70–7.92 (20H, m); *m/z* (CI) 456 ($[MH]^+$, 100%), 346 (59), 306 (50), 239

(80), 218 (47), 201 (26), 111 (60) (Found: $[MH]^+$, 456.1552. $C_{28}H_{27}NOPS$ requires $[MH]^+$, 456.1551).

With phenyl selenide

1-(Diphenylphosphinamido)-1-phenyl-4-(phenylseleno)-but-2-ene (35a), 1-(diphenylphosphinamido)-1-phenyl-2-(phenylseleno)-but-3-ene (35b) and 2-(diphenylphosphinamido)-1-phenyl-1-(phenylseleno)-but-3-ene (35c)

To a solution of diphenyl diselenide (0.68 g, 2.17 mmol) in ethanol (10 mL), under argon, at room temperature, was added $NaBH_4$ (0.16 g, 4.35 mmol) portionwise until the yellow solution became colourless. A solution of aziridine **1** (0.50 g, 1.45 mmol) in THF (5 mL) was then added, at 0 °C, and the solution was allowed to warm to room temperature before being heated under reflux overnight. The solution was then partitioned between water (10 mL) and ethyl acetate (15 mL), the aqueous layer extracted with ethyl acetate (2 × 10 mL), the organic layers washed with brine (15 mL), dried (Na_2SO_4), and filtered, and the solvent removed in vacuo to give a yellow oil which was purified by flash column chromatography on silica gel [ethyl acetate/light petroleum (1:4)] to give **35a**, **35b** and **35c** (0.53 g, 73%, colourless oil) as an inseparable mixture.

IR data for **35a**, **35b** and **35c**: R_f 0.2 [ethyl acetate/light petroleum (1:4)]; ν_{max}(film)/cm^{-1} 3344, 3060, 1210, 1124, 703.

Other data for **35a**: (≈0.06 g, 11%): δ_H (300 MHz, $CDCl_3$), 3.08 (1H, dd, J = 6.2, 10.1), 3.47 (2H, d, J = 6.8), 4.66–4.77 (1H, m, 5.52-5.67 (2H, m), 7.11–7.51 and 7.73–7.90 (20H, m).

Other data for **35b**: (≈0.20 g, 37%): δ_H (300 MHz, $CDCl_3$) 4.13–4.21 (2H, m), 4.49 (1H, ddd, J = 5.1, 10.6, 10.6) 4.80–4.91 (2H, br. m), 5.71 (1H, ddd, J = 10.1, 10.1, 16.9), 7.10–7.49, 7.72–7.88 (20H, m); δ_C (75.5 MHz, $CDCl_3$) 29.29, 56.09, 126.98, 127.31, 127.72, 127.86, 128.31, 128.40, 128.51, 128.57, 128.92, 129.19, 131.51, 131.93, 132.16, 132.22, 132.35, 134.06; m/z (CI) 504 ($[MH]^+$, 9%), 348 (36), 306 (12), 218 (74), 159 (100), 79 (52) (Found: $[MH]^+$, 504.0997. $C_{28}H_{27}NOPSe$ requires $[MH]^+$, 504.0995).

Other data for **35c**: (≈0.13 g, 25%): δ_H (300 MHz, $CDCl_3$) 3.62 (1H, dd, *J* = 6.5, 10.6), 4.01–4.09 (1H, m), 4.70 (1H, d, *J* = 3.9, 5.19-5.26 (2H, br. m), 5.86 (1H, ddd, *J* = 5.8, 10.8, 16.5), 7.08–7.50, 7.73–7.90 (20H, m).

Reference

1 Cantrill, A. A.; Jarvis, A. N.; Osborn, H. M. I.; Ouadi, A.; Sweeney, J. B. *Synlett* **1996**, 847. doi:10.1055/s-1996-5605
